# Phenotypic, Nutritional, and Antioxidant Characterization of Blanched *Oenanthe javanica* for Preferable Cultivar

**DOI:** 10.3389/fpls.2021.639639

**Published:** 2021-02-19

**Authors:** Sunjeet Kumar, Gaojie Li, Xinfang Huang, Qun Ji, Kai Zhou, Hongwei Hou, Weidong Ke, Jingjing Yang

**Affiliations:** ^1^The State Key Laboratory of Freshwater Ecology and Biotechnology, The Key Laboratory of Aquatic Biodiversity and Conservation of Chinese Academy of Sciences, Institute of Hydrobiology, Chinese Academy of Sciences, Wuhan, China; ^2^University of Chinese Academy of Sciences, Beijing, China; ^3^Institute of Vegetables, Wuhan Academy of Agricultural Sciences, Wuhan, China

**Keywords:** *Oenanthe javanica*, blanching, nutritional value, vitamins, minerals, Antioxidant capacity (AOC)

## Abstract

Blanching is a technique used in blocking sunlight for the production of tender, sweet, and delicious stems in the field. This technique is also used in water dropwort (*Oenanthe javanica*), an important vegetable in East Asia. In China, the steamed stems of water dropwort are prepared with boiled rice. However, the effect of blanching on the nutritional level and antioxidant capacity of water dropwort has not been explored yet. The current study aims to determine the nutrient contents and antioxidant capacities of five cultivars and select the best cultivar. They were mainly compared in terms of phenotypic, physiological, nutritional, and antioxidant levels after blanch cultivation. Results indicate that blanching significantly influenced the phenotype, physiology, and nutritional level of water dropwort in all cultivars. Although few parameters decreased with blanching, starch, sugars, vitamins, minerals, and antioxidant activities increased significantly in the blanched stems in mid- and post-blanching periods. The most noticeable changes were detected in post-blanching samples. Furthermore, the best cultivar (V11E0012) was identified among them. Therefore, blanched water dropwort could be consumed for achieving more nutraceuticals and antioxidants, and cultivar V11E0012 could be recommend for blanching cultivation.

## Introduction

Leafy vegetables are the key sources of dietary fibers, vitamins, proteins, carbohydrates, minerals, and macro- and micronutrients (Sarker et al., 2015a,b, 2017; Chakrabarty et al., 2018; Sarker and Oba, 2019b). These components are important sources of natural antioxidants, such as pigments (Sarker et al., 2018b), phenolics (Sarker and Oba, 2019c, 2020b,c,e; Sarker et al., 2020a,c), and flavonoids, including flavonols, flavones, flavanols, and flavanones (Sarker and Oba, 2020d,f; Sarker et al., 2020b). *Oenanthe javanica* (Blume) DC, commonly known as water dropwort, is a perennial aquatic vegetable belonging to the family Apiaceae (Jeon et al., 2007; Lee and Kim, 2009). It is mostly cultivated in freshwater, marshlands, swampy lands, canals, streams, and ditches (Minh, 2019). It is a rich source of crude fiber, vitamins, and minerals and contains adequate amounts of phenolics and flavonoids, which have excellent antioxidant properties. Owing to its nutrient quality, it is also used as a traditional Chinese medicine for treating different diseases, such as jaundice, fever, hypertension, abdominal pain, and leucorrhea (Chan et al., 2014, 2017; Jiang et al., 2015; Lu and Li, 2019). Different compounds, such as persicarin, hyperoside, and isorhamnetin, are present in water dropwort, which have different pharmacological activities, including hepatoprotective, neuroprotective, anticancer, anti-inflammatory, antioxidant, and anti-hepatitis B virus (HBV) activities. All these properties made water dropwort popular in many countries, such as China, Korea, Thailand, Japan, Malaysia, and Australia (Chan et al., 2014, 2017; Jiang et al., 2015; Lu and Li, 2019).

Owing to its distinctive aroma and taste, water dropwort is commonly used in enhancing the flavor of several dishes in East Asian countries. In Korea, water dropwort is used as an ingredient in salads or garnish in cooked items. In China, the steamed stems of water dropwort are used with boiled rice. Frying blanched water dropwort is also common in China (Ma et al., 2010; Chan et al., 2014, 2017; Bhaigyabati et al., 2017).

Blanching is a technique used in blocking the sunlight. This method stops the production of chlorophyll by inhibiting photosynthesis. Consequently, the stem of the plants becomes pale, tender, sweet, and delicious (Jishi et al., 2012; Maeda et al., 2012b; Ramu, 2012). An entire water dropwort or its parts are covered. Field blanching is used in many plants, such as white asparagus, chicory, cauliflower, and leeks (Marr, 1994; Yanping et al., 2004; Maeda et al., 2012b; Leendertz, 2019). In Japan, white asparagus is blanched mostly with the shading film and soil molding methods; the shading film method ensures tenderness and taste (Maeda et al., 2012a). Different blanching techniques, including the deep planting, deep water softening, and soil molding method, are used in processing water dropwort (Kong et al., 2005; Yuanying et al., 2008). According to Yuanying et al. (2009), the deep planting method is the most suitable for water dropwort, and the best-identified cultivars were “Yuqi shuiqin” and “Liyang shuiqin” based on yield. The effect of blanching depends on the selected method, variety of water dropwort, duration, and season (Sayre, 1929).

Information on difference in quality among water dropwort cultivars subjected to different blanching methods and which constituents contribute to nutritional value and antioxidant capacity is currently limited. Therefore, the objective of the current study is to acquire preliminary knowledge about the effects of blanching on different cultivars of water dropwort and selection of the best cultivar for blanching on the basis of nutritional profile and antioxidant capacity. We evaluated the quantity of starch, proteins, sugars, vitamins, minerals, polyphenols, flavonoids, and antioxidant capacity in different water dropwort cultivars.

## Materials and Methods

### Seedling Collection, Growth Conditions, and Experimental Design

The experiment was performed in the Institute of Aquatic Vegetables, Wuhan Academy of Agricultural Sciences, Wuhan. Five water dropwort cultivars were used, namely, V11E0100 (Liynag shuiqin), V11E0003 (Yuqi shuiqin), V11E0012 (Jianglingye Shuiqin), V11E0119 (Leping Shuiqin), and V11E0103 (TTC Shuiqin). A field with fertile soil, convenient irrigation, and drainage was used. In September, the stems of already grown water dropwort were cut into 3.3 cm long stem segments (each segment must have a stem node). Germination was promoted by placing the cut stems in a cool and ventilated place and covering them with sunshade nets for moisture retention. Water was sprinkled every day, and new shoots were germinated after a week. The newly germinated shoots were sowed in seedbeds and covered with a layer of 1–1.5 cm of soil, then a sunshade net was used to cover the seedbeds. After 30 days, when the heights of the plants were ~10 cm, small, damaged, and weak plants were removed, and the rest were planted with the hill planting method, that is, the space between hills were 10 × 10 cm, and each hill had three or four plants. When the heights of the plants were ~30 cm, blanching was applied (Kong et al., 2005; Zhu et al., 2013).

### Blanch Cultivation by the Deep Planting Method

When the water dropwort height reached up to 30 cm, the plants were removed from each hill and bound in bunches. Each bunch, containing 30 plants, was dumped in a 20 cm-deep soil, then collected after 40 days (Kong et al., 2005; Zhu et al., 2013).

The experiment was based on a factorial RCBD model. All physiological analyses were performed in triplicate for each cultivar, and each cultivar had four levels of samples as follows: Pre-blanching, Mid-blanching (After 20 days), Post-blanching (40 days), and Control.

### Phenotypic Parameters

After collection, plant height, stem length, root length, number of branches and leaves, and leaf chlorophyll were measured. SPAD-502Plus (Konica Minolta, Japan) was used in measuring chlorophyll content in the leaves. Fresh weight, dry weight, and stem moisture were also measured. Dry weight (DW) was determined after washing the stem with distilled water, drying it gently with a paper towel, and drying for 72 h at 70°C.

### Measurement of Photosynthetic Pigments

For the measurement of chlorophyll and carotenoid concentrations, 100 mg of each fresh stem was homogenized with 80% acetone and centrifuged at 7,000 × *g* for 10 min. The supernatant was collected, and absorbance (A) was measured at 663 nm for chlorophyll *a*, 646 nm for chlorophyll *b*, and 470 nm for carotenoid with an ELISA plate reader (i3x molecular devices, USA; Sarker and Oba, 2020d). The concentrations of chlorophyll and carotenoids were calculated as follows: chlorophyll *a* = 12.21(A_663_) – 2.81(A_646_), chlorophyll *b* = 20.13(A_646_) – 5.03(A_663_), total chlorophyll = chlorophyll *a* + chlorophyll *b*, and carotenoids = [1,000(A_470_) – 3.27(chl *a*) - 104(chl *b*)] /229, where A_646_ = absorbance at a wavelength of 646 nm, A_663_ = absorbance at a wavelength of 663 nm, and A_470_ = absorbance at a wavelength of 470 nm.

### Assays for Total Protein Content, Total Amino Acids, and Total Antioxidant Capability

Approximately 200 mg of fresh stem from each sample was homogenized with 1,800 μL of PBS (pH 7.4, 0.1 M) with a glass homogenizer, then centrifuged at 3,500 × *g* for 10–12 min. The supernatant was used in determining total proteins, total amino acids, and antioxidant capacity.

The Coomassie brilliant blue method was used in determining total protein content with commercial total protein assay kit (A045-2) made by Nanjing Jiancheng Bioengineering Institute, China, and absorbance was measured at 595 nm. Total amino acid content was determined also with a commercially available test kit (A026-1-1, Nanjing Jiancheng Bioengineering Institute, Nanjing, China). Copper ions were reacted with various amino acids for the production of blue complexes. Absorbance at a certain wavelength stands was directly proportional to total amino acid content. Absorbance was measured at 650 nm.

Various antioxidative compounds can reduce Fe^3+^ to Fe^2+^, and Fe^2+^ can react with phenanthroline to produce a stable complex, which can be quantified at a certain wavelength. Total antioxidant capability was determined with the commercial total antioxidative capability assay kit (A015-1) of Nanjing Jiancheng Bioengineering Institute, China, and absorbance was measured at 520 nm (Hussain et al., 2016; Dai et al., 2018; Yang et al., 2018).

### Determination of Starch

Starch determination was performed using a commercially available test kit of Nanjing Jiancheng Bioengineering Institute, Nanjing, China was used. Approximately 50 mg of fresh samples of each stem were homogenized with 500 μL of reagent I (provided by the company) and centrifuged three times at 8,000 × *g* for 15 s with an interval of 30 s. The mixture was boiled at 80°C for 20 min and centrifuged at 4,000 × *g* for 5 min. The supernatant was discarded, and the pellet was diluted with 250 μL of ddH_2_O, then boiled at 90°C for 15 min. Approximately 175 μL of reagent II was added and incubated at 25°C for 15 min. Furthermore, 425 μL of ddH_2_O was added and centrifuged at 4,000 × *g* for 10 min. The supernatant was collected, which was processed further according to the protocol provided by the company. Finally, absorbance was measured at 620 nm for starch quantification (Tang et al., 2019).

### Determination of Soluble Sugars

For the analysis of soluble sugars, 0.1 g of stem samples were homogenized in 1 mL of double distilled water with a glass homogenizer. The tubes were boiled at 95°C for 10 min and cooled with tap water. After cooling, the homogenate was centrifuged at 4,500 × *g* for 10 min. Then, the supernatant was diluted with double distilled water (1:9). The diluted extracts were used in determining soluble sugars with a commercially available test kit (A145-1-1, Nanjing Jiancheng Bioengineering Institute, Nanjing, China). Finally, absorbance was measured at 620 nm for soluble sugar quantification (Tang et al., 2019).

### Determination of Total Polyphenols, Flavonoids, and DPPH

For this purpose, fresh stem was freeze-dried in a lyophilizer for 48 h. After lyophilization, 250 mg of freeze-dried samples were used for analysis. Samples were crushed to the fine powder in mortar and pestle and homogenized with 16 mL of 10 mM methanol/ammonium acetate 50:50 (v/v). After that, homogenates were placed in an ultrasound bath for 15 min, centrifuged at 10,000 × *g* for 15 min. Supernatant was collected and filtered through 0.45 μm nylon filter. The filtered supernatant was used for further analysis (Santos et al., 2014).

### Determination of Total Polyphenols

Gallic acid was used as a standard for the quantification of total polyphenols. For the 100 ppm stock solution preparation, 1 mg of gallic acid was dissolved in 10 mL of distilled water. Then working solutions of 80, 60, 40, 20, 10, 5, and 2.5 ppm were prepared and used to quantify total polyphenols.

For the determination of total polyphenols, Folin-Ciocalteu method was used with some modifications given by Sarker and Oba (2020a). Five hundred microliters of plant extract was mixed with 2.5 mL Folin-Ciocalteu reagent (1:10), then 2 mL 0.75 g/mL Na_2_CO_3_.10H_2_O solution was added, the mixture was incubated at 45°C for 15 min. After that, the mixture was placed at room temperature for 1 h (samples covered in aluminum foil to avoid exposure to light). Finally, the absorbance was measured at 760 nm, and the results were expressed as GAE/100 g.

### Determination of Flavonoid Content

Catechin was used as a standard for the quantification of flavonoids. Preparation of the stock solution, and working solutions, and standards were the same as mentioned for polyphenols.

For the determination of flavonoids, aluminum chloride method was used described by Sarker and Oba (2019a) and Barroso et al. (2011). For this purpose, 4 mL distilled water was mixed with 300 μL NaNO_2_ solution (0.5 g/mL), then 1 mL of plant extract was added. After 5 min, 300 μL AlCl_3_ (1 g/mL) solution, 2 mL NaOH (1 mol/L), and 2.4 mL double distilled water were added simultaneously. Finally, the absorbance level was measured at a wavelength of 510 nm, and the results were analyzed against standard CE/100 g.

### DPPH Scavenging Activity

DPPH (2,2-diphenyl-1-picryl-hydrazyl-hydrate) scavenging activity in a freeze-dried stem was determined with the method described by Sarker and Oba (2020a) with some modifications. Approximately 5 mL of DPPH (0.1 mM in methanol) was mixed with 2 mL of plant extract. The tube was vortexed and incubated for 30 min at 25°C. Finally, the decolorization of DPPH (dark purple) was determined at 517 nm. Initially, a dark purple blank DPPH solution without extract was measured. Results were determined against gallic acid, and percent inhibition was measured with the following correlation:

$$\%\text{Inhibition=}\frac{\left(\text{Ablank-Asample}\right)}{\text{Ablank}}\times \text{100}$$

### Determination of Vitamins

For the determination of vitamins A, B1, and B2, 1 g of fresh samples of each stem were homogenized with 9 mL of PBS (pH 7.4, 0.01 M) with a glass homogenizer. The homogenates were further lysed with a sonicator for 30–60 s, then centrifuged at 5,000 × *g* for 10 min. The supernatant was collected for further analysis.

The supernatant was used for vitamin A, B1, and B2 analyses with commercially available ELISA kits (Shanghai Jingkang Bioengineering Co. Ltd. © 2017 gelatins.com.cn). The protocols for vitamin A, B1, and B2 were the same. Each kit had its own 96 wells, precoated microwell plates, standards, and chemicals. Each sample (50 μL) was added to the microplate wells precoated with antibodies. After the addition of the samples, the microplates were gently mixed and incubated for 45 min at 37°C. The liquid was discarded, and each well was washed four times with 350 μL of washing solution and dried on absorbent paper. Approximately 50 μL of biotinylated anti-IgG was added and incubated for 30 min at 37°C, then the washing step was again performed. After washing, 50 μL of streptavidin-HRP was added to each well, which was incubated at 37°C for 15 min. The washing step was repeated, and 50 μL of chromogen solution A and chromogen solution B were added stepwise. The mixtures were incubated at 37°C for 15 min. After incubation, 50 μL of termination solution was added to each well. Finally, OD was determined at 450 nm.

For vitamin C analysis, 50 mg of fresh samples of each stem were homogenized with 450 μL of PBS (pH 7.4, 0.1 M) with a glass homogenizer and then centrifuged at 4,500 × *g* for 10–12 min. The supernatant was used in determining vitamin C with a commercially available test kit (A009, Nanjing Jiancheng Bioengineering Institute, Nanjing, China). Approximately 150 μL of the supernatant was mixed with 450 μL of reagent I (available in the kit), left to stand for 15 min at room temperature, and centrifuged at 4,000 × *g* for 10 min. The supernatant was then collected and processed further with the reagents of the kit, and absorbance was measured at 536 nm.

### Mineral Determination

For the determination of ions (Na, K, Mg, Ca, Fe, Cu, Mn, and Zn contents), 100 mg of dried samples of each stem were digested with 6 mL of nitric acid with a microwave digestion system (Multiwave 3000, Anton Paar, Austria) for 1.5 h. The digested samples were diluted to up to 10 mL with ultra-deionized water. Minerals (Na, K, Mg, and Ca) were determined by inductively coupled plasma-atomic emission spectroscopy (ICP-OES; Optima8000, PerkinElmer USA). Meanwhile, Fe, Cu, Mn, and Zn contents were measured by inductively coupled plasma mass spectrometry (ICP-MS; NexION300X, PerkinElmer USA) at the public technical service center in the Institute of Hydrobiology, Chinese Academy of Sciences, Wuhan (Colomer-Winter et al., 2018; Kumar et al., 2020).

### Statistical Analysis

All the measurements were performed in triplicate, and SPSS 25.0 statistical program (IBM Crop. Armonk, NY, USA) was used in statistical analysis. Tukey tests were performed to determine significant differences (*P* ≤ 0.05) among treatments. GraphPad Prism 7 (San Diego, California, USA) was used for figures, and significant differences were indicated by different alphabets. All the data were represented as mean ± standard error (S.E).

## Results

### Phenotypic Results

**Figure 1:**
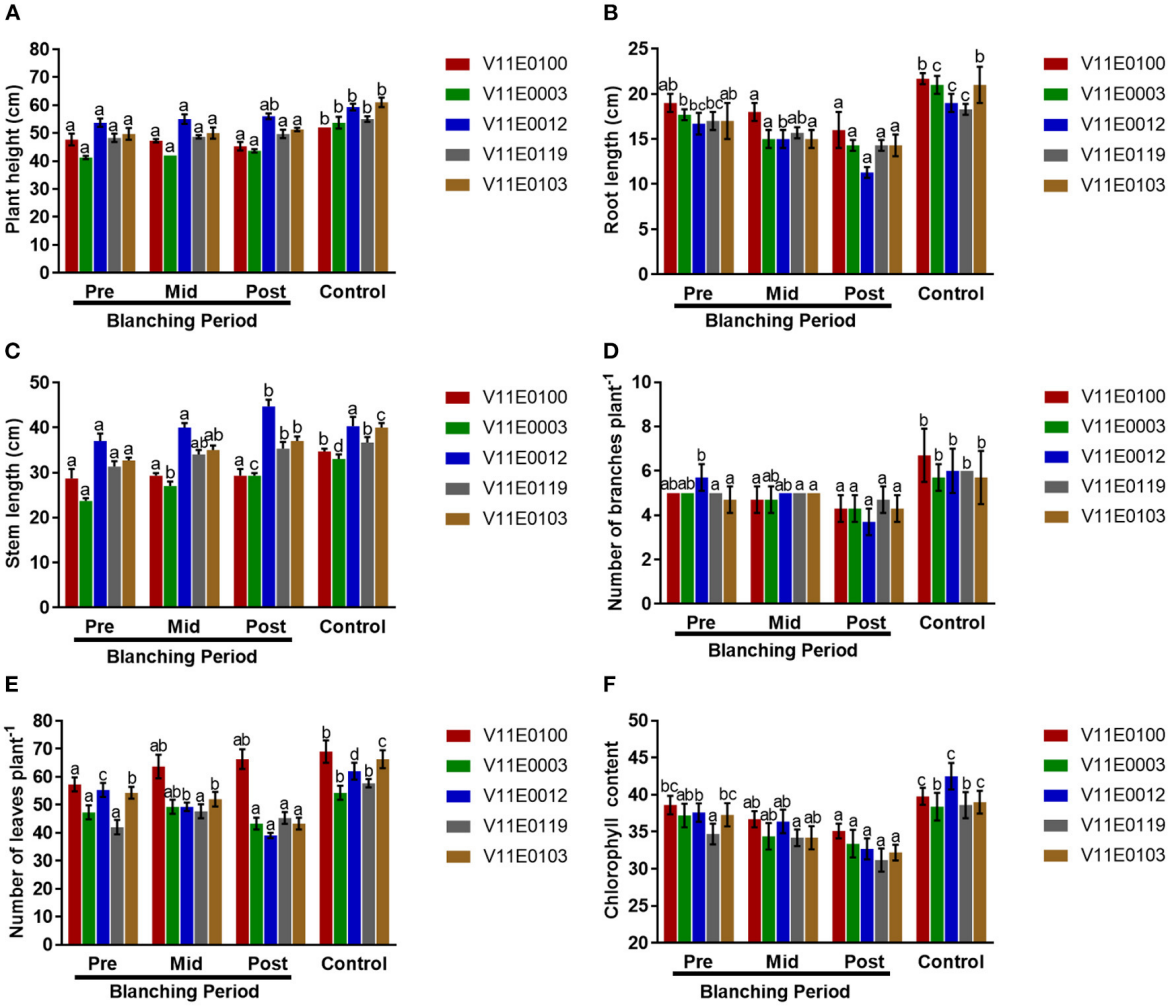
Effect of blanching on the phenotype of five water dropwort cultivars. **(A)** Plant height, **(B)** root length, **(C)** Stem Length, **(D)** Number of branches, **(E)** Number of leaves, and **(F)** Chlorophyll content of leaves. Four types of samples were used for analysis; Pre-blanching, Mid-blanching (20 days), Post-blanching (40 days), and Control (grown under normal conditions). Different letters indicate a significant difference (*P* < 0.05) among the treatments according to the Tukey test. Values are means ± SE.

To explore the effects of blanching on the different cultivars of water dropwort, we first analyzed the phenotypes under different treatments. Plant growth properties were influenced by “mid-blanching” and “post-blanching” in all cultivars, and comparatively more changes were detected in the post-blanching samples (Figure 1, Supplementary Figure 1).

The plant heights of all cultivars slightly increased mid-blanching and post-blanching, but no significant difference was detected in comparison with the pre-blanching samples (*P* < 0.05). Similarly, the plant height of each sample grown under normal conditions (control) was significantly higher than that of its counterpart (Figure 1A).

Stem length and root length increased and decreased, respectively, with blanching period in all the cultivars, and clear difference in length was observed between the post-blanching and pre-blanching samples (*P* < 0.05). The maximum reduction in root length was observed in the V11E0012 cultivar after the blanching period (Figure 1B). The maximum post-blanching lengths of the stems of V11E0003 and V11E0012 were 23.6 and 20.8% higher, respectively, than those of their pre-blanching counterparts (Figure 1C). By contrast, root and stem lengths were significantly increased in the samples grown under control conditions compared with their counterparts.

The number of branches decreased throughout the blanching period compared with that in the control. The post-blanching samples had a lower number of branches than their counterparts (*P* < 0.05). Moreover, V11E0012 showed the least number of branches, showing a 35.1% decrease in the number of branches after blanching relative to the number before blanching (Figure 1D).

Different trends were observed in different cultivars in terms of the number of leaves. The number of leaves in three cultivars (V11E0003, V11E0012, and V11E0103) was reduced during the blanching period. The least number of leaves was detected in V11E0012 and V11E0103 by 29.5 and 20.2%, respectively, in the post-blanching samples (*P* < 0.05). Interestingly, the number of leaves of V11E0100 and V11E0119 subjected to blanching was 15.7 and 7.85% higher, respectively, than the pre-blanching samples. Furthermore, the control samples showed a significantly higher number of leaves than their counterparts (Figure 1E).

Reduction in chlorophyll content in the leaves was observed throughout the blanching period, whereas samples grown under control conditions showed an increment in chlorophyll content. Moreover, chlorophyll content in the post-blanching samples was significantly lower than that in their counterparts (*P* < 0.05; Figure 1F).

**Figure 2:**
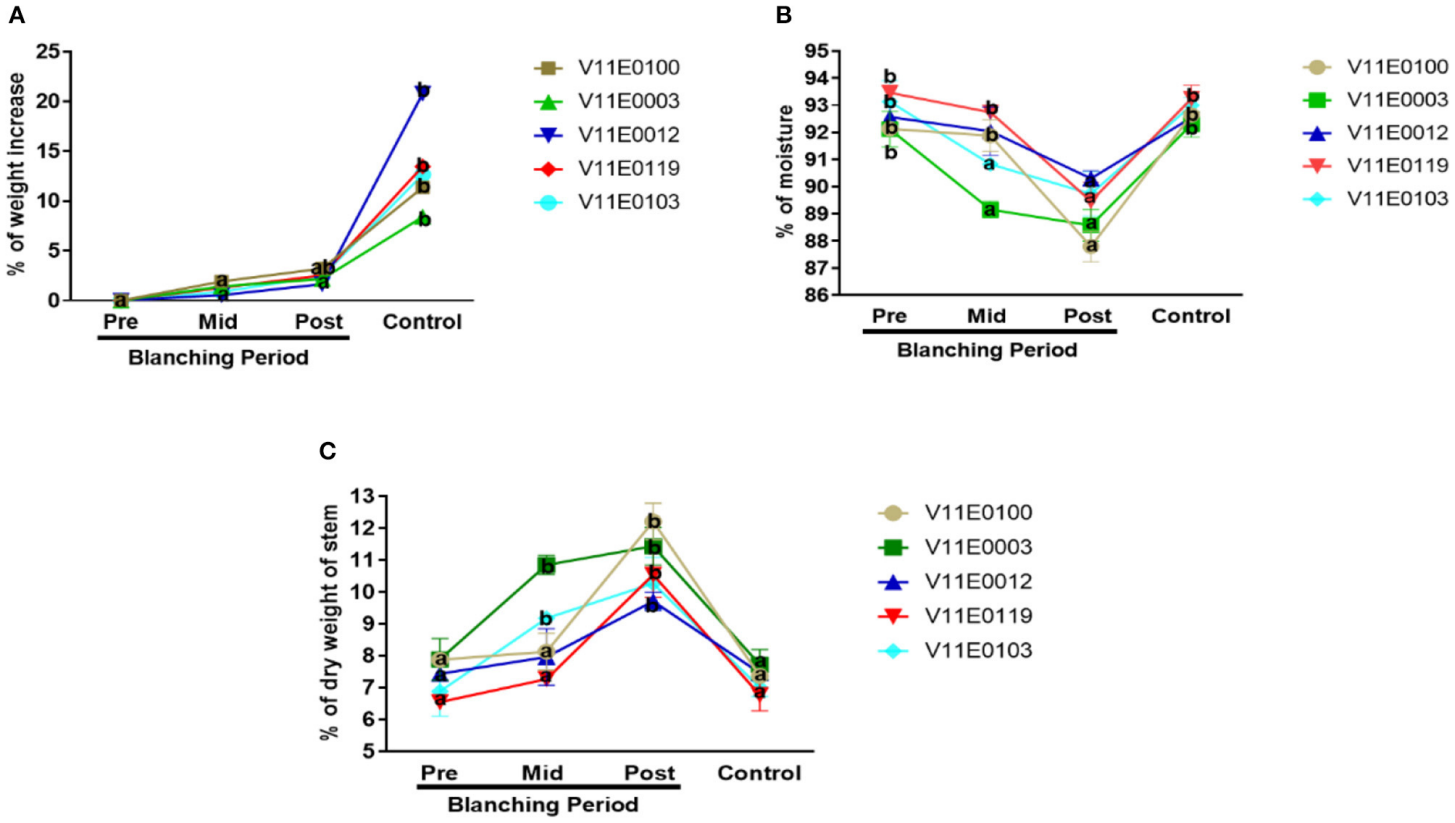
Effect of blanching on the **(A)** fresh biomass, **(B)** moisture content, and **(C)** dry weight of the stem in five water dropwort cultivars. Four types of samples were used for analysis; Pre-blanching, Mid-blanching (20 days), Post-blanching (40 days), and Control (grown under normal conditions). Different letters indicate a significant difference (*P* < 0.05) among the treatments according to the Tukey test. Values are means ± SE.

The fresh biomass of the stems increased significantly in the control samples relative to that in the pre-blanching samples (*P* < 0.05). By contrast, an insignificant increase during the blanching period was observed in the stem biomass of all the cultivars. The maximum increment after blanching was detected in V11E0100 (3.22%) as compared to its pre-blanching counterpart (Figure 2A).

Reduction in moisture content and increment in dry weight percentage in the stems were detected throughout the blanching period, and highly significant difference was found between the post-blanching samples and their counterparts (*P* < 0.05). Moreover, V11E0100 and V11E0003 showed larger differences than the control and pre-blanching samples. However, no significant difference in the percentage of moisture content and dry weight of stem was present in samples grown under control conditions and pre-blanching samples (Figures 2B,C).

### Physiological Parameters

#### Photosynthetic Pigments

**Figure 3:**
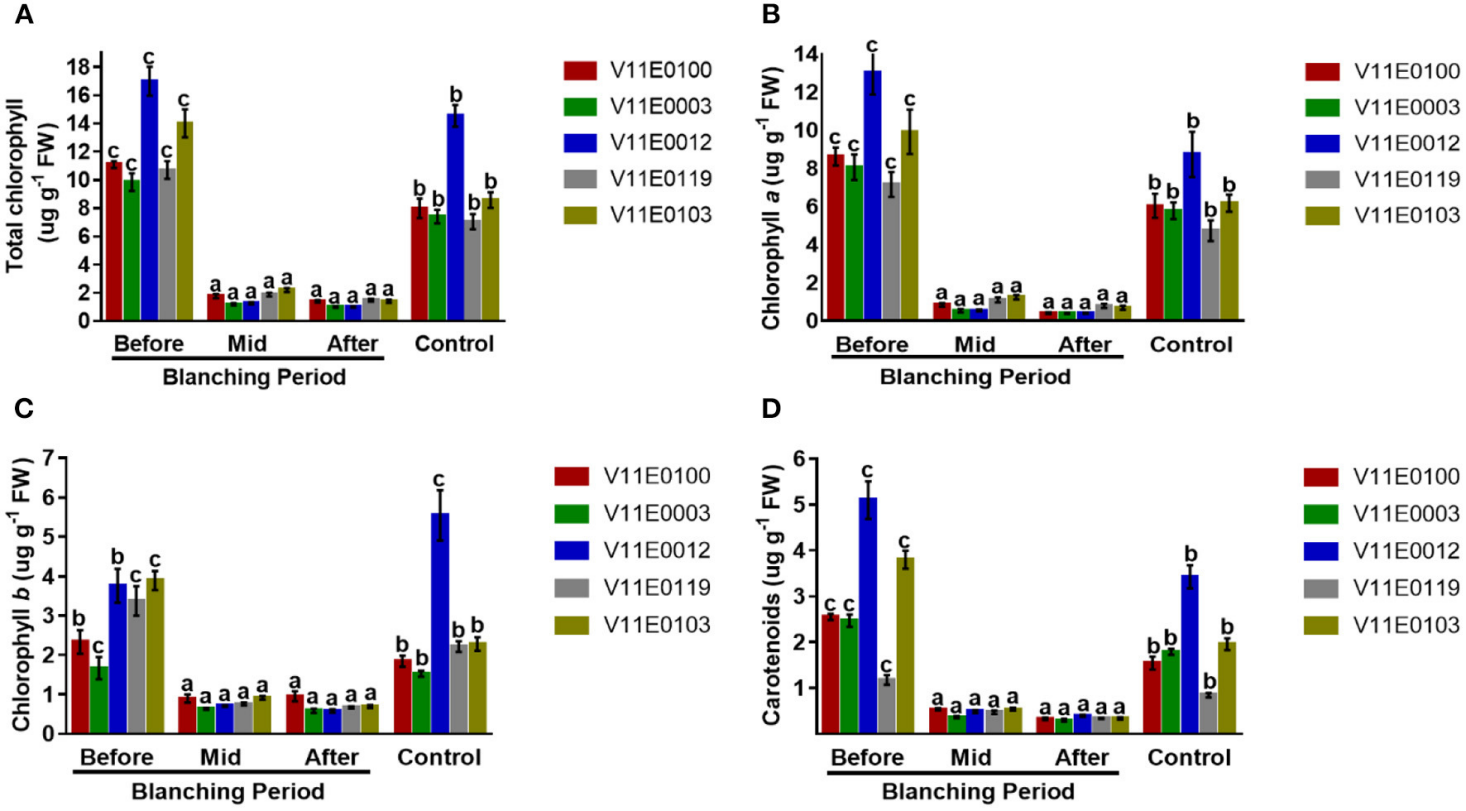
Effect of blanching on the photosynthetic pigments of the stem in five water dropwort cultivars. **(A)** Total chlorophyll content, **(B)** chlorophyll *a* content, **(C)** chlorophyll *b* content, and **(D)** carotenoids concentration. Four types of samples were used for analysis; Pre-blanching, Mid-blanching (20 days), Post-blanching (40 days), and Control (grown under normal conditions). Different letters indicate a significant difference (*P* < 0.05) among the treatments according to the Tukey test. Values are means ± SE.

Blanching has a drastic effect on stem photosynthetic pigments of the stem (Figure 3). Total chlorophyll content and chlorophyll *a*, chlorophyll *b*, and carotenoid contents decreased significantly throughout the blanching period, and maximum difference was detected in the post-blanching samples in comparison with the pre-blanching and control samples (*P* < 0.05). Although chlorophyll *b* decreased during the blanching period, the ratio of chlorophyll *b*/*a* increased during the blanching period compared with the ratios in the control and pre-blanching samples (Figure 3).

#### Nutritional Parameters

Nutritional parameters, including minerals, vitamins, starch, sugars, proteins, amino acids, secondary metabolites, and antioxidant capability were influenced during the blanching period in all the cultivars.

### Total Protein Content

**Figure 4:**
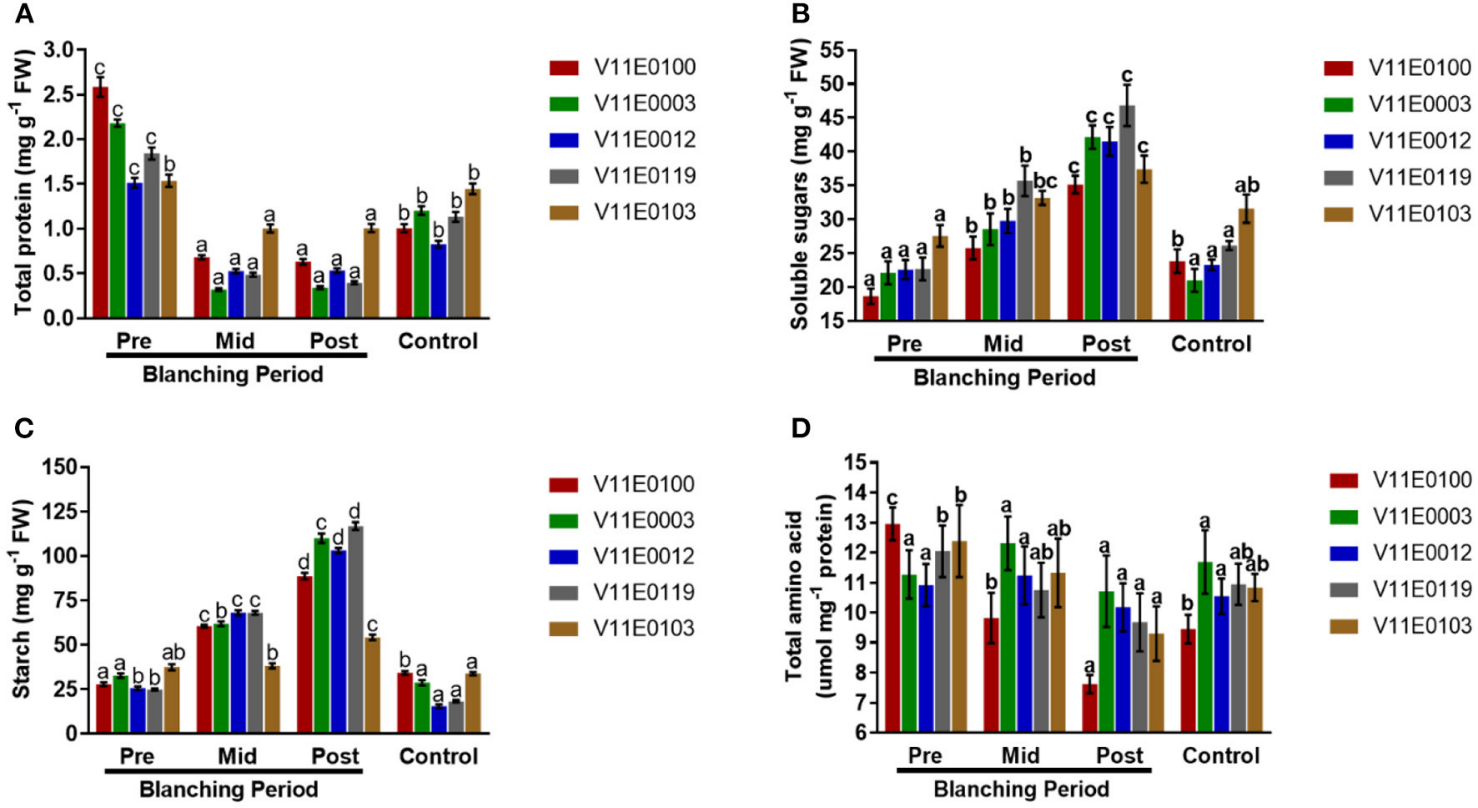
Effect of blanching on the **(A)** total protein content, **(B)** soluble sugars content, **(C)** starch content, and **(D)** total amino acid content in the stem of five water dropwort cultivars. Four types of samples were analyzed; Pre-blanching, Mid-blanching (20 days), Post-blanching (40 days), and Control (grown under normal conditions). Different letters indicate a significant difference (*P* < 0.05) among the treatments according to the Tukey test. Values are means ± SE.

Blanching showed a negative effect on the total protein content in the stems of all the cultivars (Figure 4A). The least protein content was detected in the post-blanching samples (*P* < 0.05). Moreover, V11E0103 and V11E0012 were comparatively less affected by blanching, and their protein contents after blanching were 34.5 and 64.7% compared to pre-blanching samples (Figure 4A).

### Soluble Sugars Content

The contents of soluble sugars in the stems of all the cultivars increased during the blanching period, and a highly significant difference was found between the post-blanching samples and comparison to other counterparts (*P* < 0.05). Moreover, the soluble sugar contents of V11E0119, V11E0003, and V11E0012 significantly increased compared with the other cultivars (Figure 4B).

### Starch Content

A significant increment in starch content was observed in the stems during the blanching period, and the post-blanching samples showed a maximum increase compared with their counterparts (*P* < 0.05). The starch contents in V11E0119, V11E0003, and V11E0012 were high relative to those in their counterparts. By contrast, a decrease in the control samples was observed in comparison with pre-blanching samples, except V11E0012 (Figure 4C).

### Total Amino Acids

Total amino acids were reduced during the blanching period in the stems of all the cultivars, and the least content was detected in the post-blanching samples (*P* < 0.05). Furthermore, a minute reduction in amino acids content was observed in post-blanching samples of V11E0003 and V11E0012 (4.9 and 6.8%) (**Figure 4D**).

### Polyphenol and Flavonoid Content Analysis

**Figure 5:**
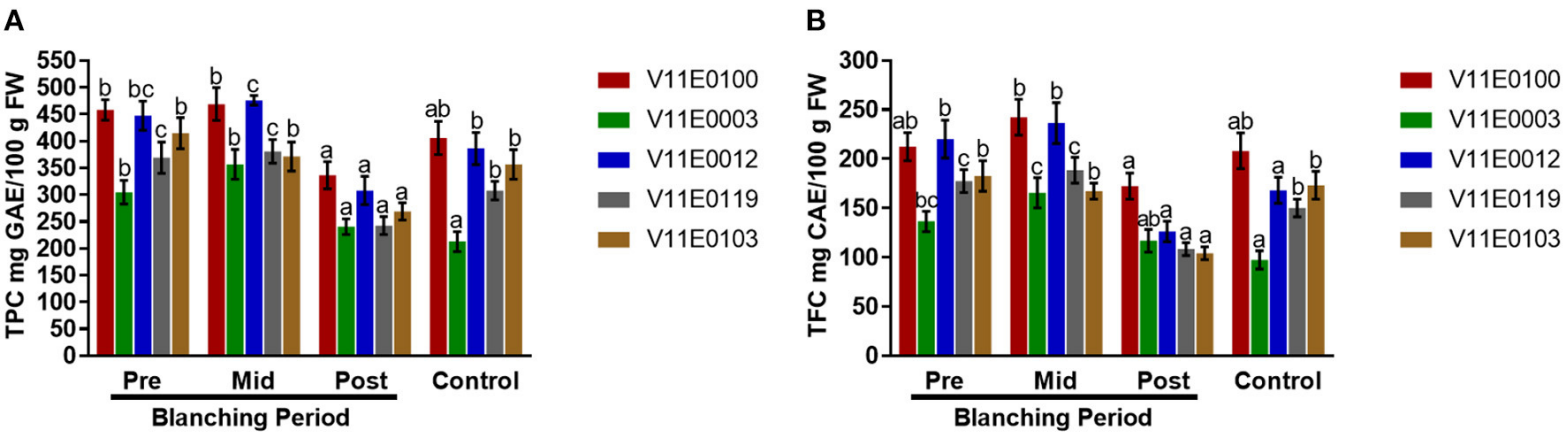
Effect of blanching on the total polyphenols and flavonoids content in the stem of five water dropwort cultivars. **(A)** Total polyphenols content, and **(B)** total flavonoids content. Four types of samples were analyzed; Pre-blanching, Mid-blanching (20 days), Post-blanching (40 days), and Control (grown under normal conditions). Different letters indicate a significant difference (*P* < 0.05) among the treatments according to the Tukey test. Values are means ± SE.

Polyphenols and flavonoids showed different trends during blanching. The contents of polyphenols and flavonoids increased in the mid-blanching period, but decreased in the post-blanching samples compared with the pre-blanching samples, except V11E0103, which showed a constant decline at in mid- and post-blanching periods (Figures 5A,B). Moreover, V11E0100 and V11E0012 were found comparatively less affected by blanching (*P* < 0.05). The control samples of all the cultivars showed reduced polyphenol and flavonoid contents compared with the pre-blanching samples (Figures 5A,B).

### DPPH Scavenging Activity

**Figure 6:**
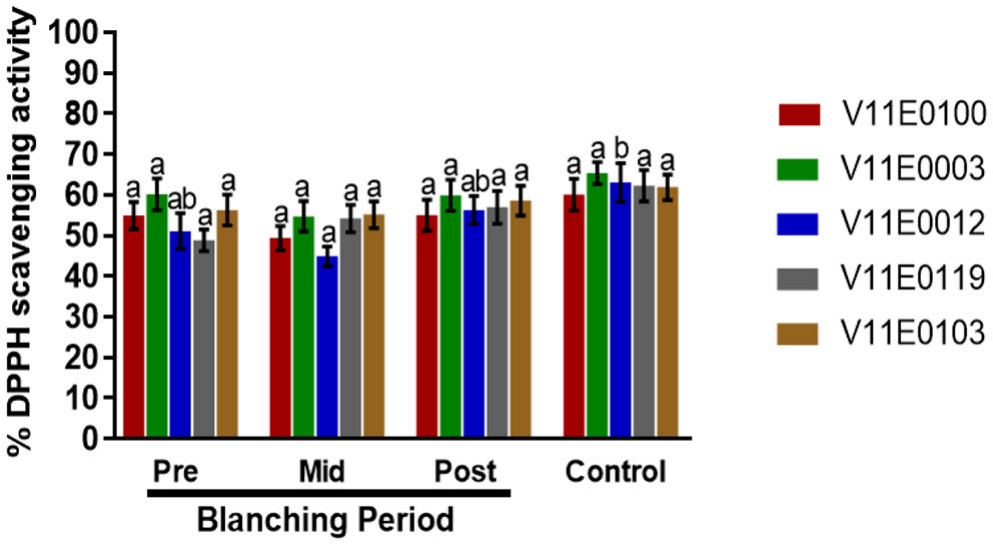
Effect of blanching on the DPPH (2,2-diphenyl-1-picryl-hydrazyl-hydrate) activity in the stem of five water dropwort cultivars. Gallic acid was used as a standard. Four types of samples were analyzed; Pre-blanching, Mid-blanching (20 days), Post-blanching (40 days), and Control (grown under normal conditions). Different letters indicate a significant difference (*P* < 0.05) among the treatments according to the Tukey test. Values are means ± SE.

DPPH (2,2-diphenyl-1-picryl-hydrazyl-hydrate) scavenging activity in the stems of all the cultivars showed no significant change in the mid-blanching period (Figure 6). A minor increase was detected in each cultivar at the post-blanching period. Moreover, V11E0119 and V11E0012 showed 16.7 and 10.1% increase in DPPH activity, respectively, in the post-blanching samples compared with the pre-blanching samples (Figure 6).

### Total Antioxidant Capacity

**Figure 7:**
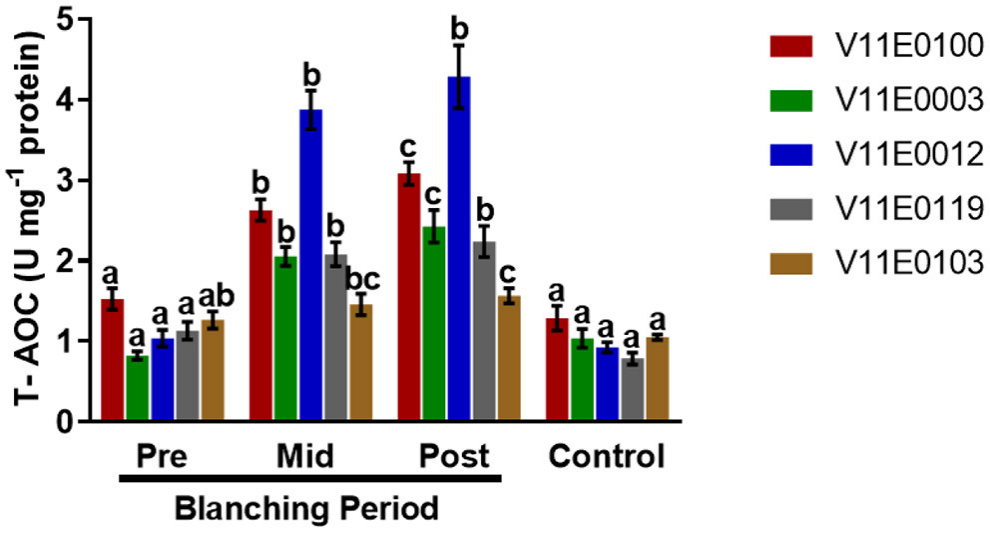
Effect of blanching on the total antioxidant capacity (T-AOC) in the stem of five water dropwort cultivars. Four types of samples were analyzed; Pre-blanching, Mid-blanching (20 days), Post-blanching (40 days), and Control (grown under normal conditions). Different letters indicate a significant difference (*P* < 0.05) among the treatments according to the Tukey test. Values are means ± SE.

A significant increase in total antioxidant capacity was observed in the stems during the blanching period, and post-blanching samples showed the highest increase (*P* < 0.05). The highest total antioxidant capacities were detected in V11E0012 and V11E0100 (Figure 7).

### Vitamins Analysis

The concentrations of vitamins A, B1, B2, and C significantly increased in the stems of the five cultivars during the blanching period, and the post-blanching samples showed the highest increments compared with their counterparts (*P* < 0.05).

**Figure 8:**
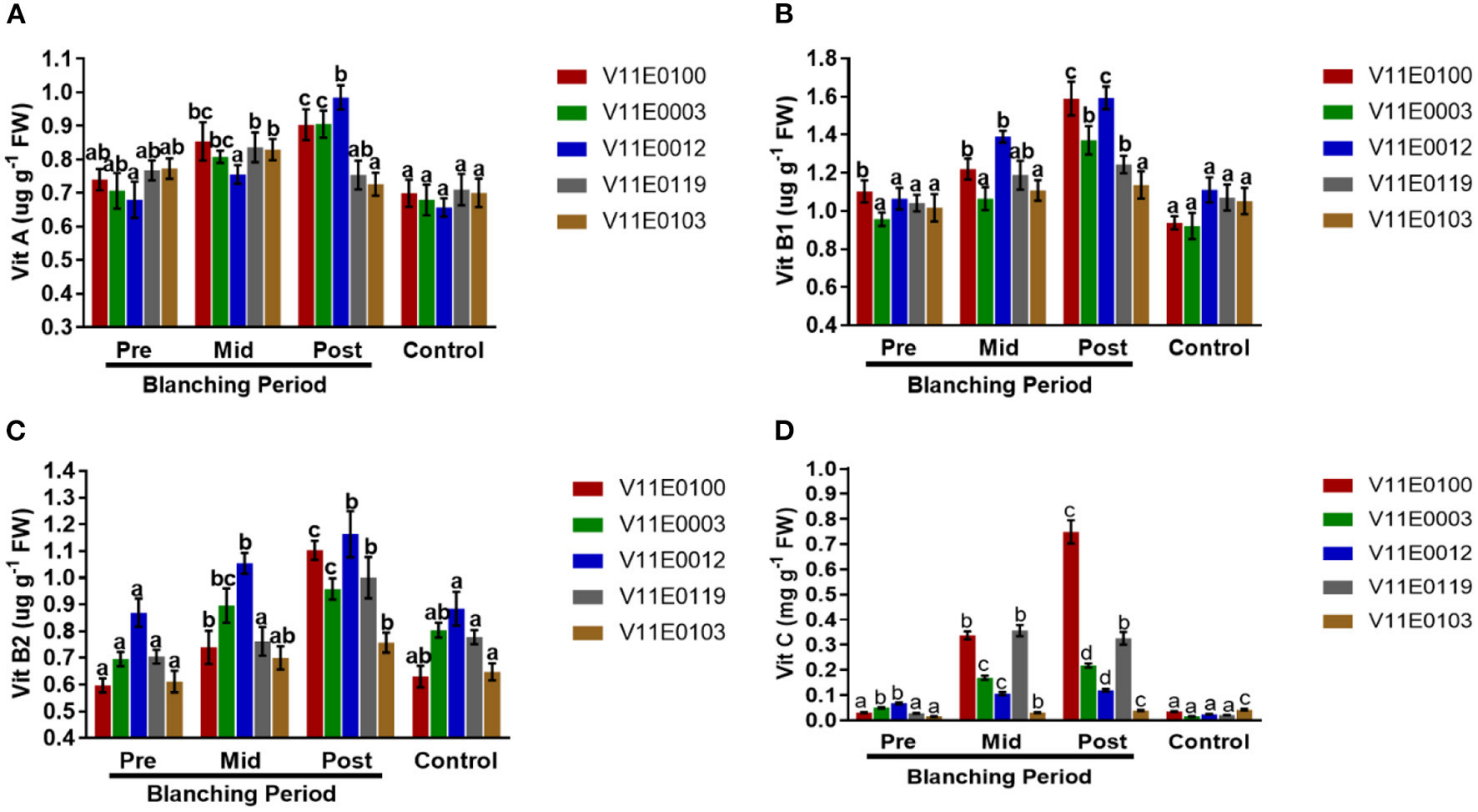
Effect of blanching on the content of vitamins in the stem of five water dropwort cultivars. **(A)** Vitamin A content, **(B)** Vitamin B1 content, **(C)** Vitamin B2 content, and **(D)** Vitamin C content. Four types of samples were analyzed; Pre-blanching, Mid-blanching (20 days), Post-blanching (40 days), and Control (grown under normal conditions). Different letters indicate a significant difference (*P* < 0.05) among the treatments according to the Tukey test. Values are means ± SE.

The highest content of vitamin A was detected in the post-blanching samples of V11E0012 and V11E0003. However, V11E0103 and V11E0119 showed minor decreases in vitamin A content after blanching (Figure 8A). The contents of vitamin B1 and B2 were higher in the post-blanching samples of V11E0012 and V11E0100 than in their pre-blanching counterparts (Figures 8B,C).

Similarly, the content of vitamin C was higher in the post-blanching samples of V11E0100 and V11E0119 compared with other counterparts. By contrast, the control samples of V11E0003, V11E0012, and V11E0119 showed lower vitamin C contents than their pre-blanching samples (Figure 8D).

### Mineral Analysis

**Figure 9:**
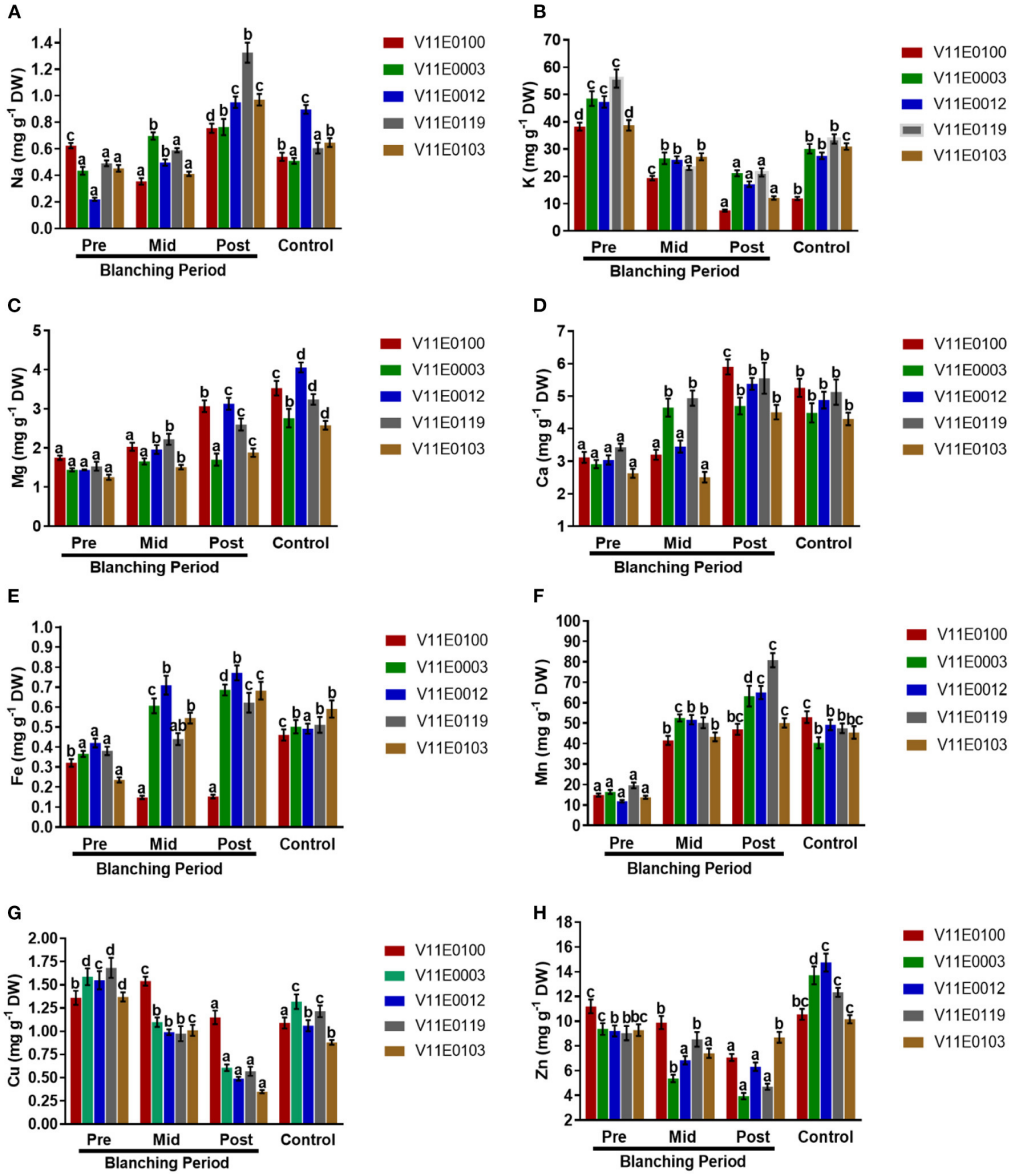
Effect of blanching on the uptake of Minerals. **(A)** Na uptake, **(B)** K uptake, **(C)** Mg uptake, **(D)** Ca uptake, **(E)** Fe uptake, **(F)** Mn uptake, **(G)** Cu uptake, and **(H)** Zn uptake in the stem of five water dropwort cultivars. Four types of samples were analyzed; Pre-blanching, Mid-blanching (20 days), Post-blanching (40 days), and Control (grown under normal conditions). Different letters indicate a significant difference (*P* < 0.05) among the treatments according to the Tukey test. Values are means ± SE.

Na uptake rate and K content increased and decreased, respectively, in the stems of all the cultivars during the blanching period, and the highest significant difference was observed between the post-blanching samples and their counterparts (*P* < 0.05). Moreover, V11E0119 and V11E0012 showed the highest Na uptake rates. However, the lowest K uptake rates were observed in the post-blanching samples of V11E0100 and V11E0103 (Figures 9A,B).

Samples grown under control conditions showed significant increases in the uptake rates of Mg and Ca ions in the stems of all the cultivars compared with the pre-blanching samples. Similarly, Mg and Ca increased during the blanching period, and a significant difference between the post-blanching and pre-blanching samples was observed (*P* < 0.05). The Mg and Ca uptake rates in the V11E0012 and V11E0100 post-blanching samples were higher than those in the pre-blanching samples of the same cultivars (Figures 9C,D).

An increment in uptake of Fe and Mn contents in the stem of all cultivars were detected during the blanching period and in plants grown under control conditions (*P* < 0.05). The post-blanching samples showed the highest uptake rates for ions compared with their counterparts, except V11E0100, which showed a decrease in the uptake of Fe during the blanching period. V11E0012 and V11E0003 showed comparatively good Fe and Mn rates (Figures 9E,F).

Reduction in the uptake of Cu and Zn was observed throughout the blanching period. The lowest uptake was detected in the post-blanching samples (*P* < 0.05). Moreover, during the blanching period, the effect on Zn and Cu uptake was low in V11E0103 and V11E0100, respectively (Figures 9G,H).

## Discussion

### Effect of Blanching on Phenotypic Parameters and Selection of Suitable Cultivar for Plant Growth, Particularly Stem Biomass Under Blanching Conditions

Differences among the blanching methods were found by previous studies, and the deep planting method is the best method for the blanching of water dropwort (Yuanying et al., 2008). In the current study, we used the deep planting method on the five water dropwort cultivars. Stem length and fresh and dry biomass increased, whereas root length decreased during the blanching. Under submerged conditions, shoot elongation was observed to be a response to shade and flooding in *Arabidopsis* and rice (Colebrook et al., 2014; Kuroha et al., 2018). Singh et al. (2014) reported that different rice cultivars showed different responses toward submergence. In some cultivars, shoot length increased, whereas root length decreased. This increase or elongation was due to increase in gibberellin biosynthesis and ethylene signaling. Ethylene reduces the content of abscisic acid and increases the level of gibberellin, thereby promoting the growth of plants under these conditions (Choi, 2007; Colebrook et al., 2014; Kuroha et al., 2018). Other responses to shade include increased stem growth, leaf hyponasty, and petiole extension. Moreover, the actions of various hormones, such as auxin, ethylene, gibberellin, and brassinosteroids are also involved (Keuskamp et al., 2010; Stamm and Kumar, 2010; Colebrook et al., 2014). We assume that increase in the stem length of blanched water dropwort is essential to its adaptation to different environmental conditions. The roles of various hormones in water dropwort subjected to blanching should be studied.

According to Girault et al. (2008), dark conditions cause a reduction in the number of leaves and bud burst in *Rosa* species. Similarly, the number of leaves of *Eucalyptus globulus* decreases under reduced light intensity (James and Bell, 2000). In the current study, the number of branches decreased during blanching. Jackson et al. (1987) and Ella et al. (2003) mentioned that the production and accumulation of ethylene under stress conditions promote stem elongation and initiate leaf senescence and subsequently reduce the rate of photosynthetic carbon fixation during deep planting, flooding, and submergence. The level of leaf chlorosis increases during submergence (Jackson et al., 1987; Ella et al., 2003).

Therefore, through phenotypic study, we showed that overall plant growth and particularly stem biomass of V11E0012 during blanching increased significantly. Since it is known that the stem of water dropwort is used as food.

### Effect of Blanching on Photosynthetic Pigments

Blanching has a direct effect on chlorophyll content, and we found a decrease in chlorophyll content in the leaves and stems of water dropwort samples subjected to blanching. We also found an increase in chlorophyll *b*/*a* ratio in the stems. Zhang et al. (2018) mentioned that blanching intensely reduces the chlorophyll content and consequently improves the flavor and exterior quality of water dropwort. In general, chlorophyll content decreases under low light stress. *Brassica campestris* shows a reduction in the contents chlorophyll (t. chl, chl *a*, and chl *b*) and carotenoids, whereas, increment in chlorophyll *b*/*a* ratio is observed under low light intensity (Zhu et al., 2017).

Reduction in the photosynthetic capacity of blanched water dropwort might be due to decreased photosynthetic rate, stomatal conductance, intracellular CO_2_ concentration, and transpiration rate (Sarker and Oba, 2018b,c, 2020g). Jackson et al. (1987), Ella et al. (2003), and Singh et al. (2014) mentioned that increase in the rate of chlorophyll loss in leaves is caused by ethylene, which upregulates the expression of genes and enzyme activity of chlorophyllase, which causes the breakdown of chlorophyll.

### Effect of Blanching on Nutritional and Antioxidant Capacity

Proteins are required in biological activities and respond to environmental stress. Noticeable effects on the compositions and contents of proteins are observed under biotic and abiotic stresses (Zhu et al., 2017). In the current study, we found that the protein content of a blanched stem was significantly lower than in the protein contents of pre-blanched and control samples. This result shows that protein accumulation is inhibited under dark and low light conditions, and this inhibition leads to decreased production. Pacholczak et al. (2005) showed that etiolation and shading conditions significantly reduce protein content in *Cotinus coggygria*. Similarly, Zhu et al. (2017) mentioned that low light can influence protein content in *Brassica campestris*.

The blanching of water dropwort stems enhances sugar content (Sun and Liu, 2008). In our study, the lowest soluble sugar content was observed under pre-blanching and control conditions, and soluble sugar content was responsive to blanching. These results are consistent with a previous report. Moreover, V11E0119 and V11E0012 showed higher sugar contents throughout the blanching period than their counterparts. Soluble sugars are essential carbon sources and osmoregulators of plant growth, and the levels of soluble sugars reflect the nutritional status of plants (Shao et al., 2014). Stress disrupts metabolic activity and decreases ATP production, thereby hampering the growth and decreases further vegetative growth of plants (de Oliveira and Joly, 2010). This effect is usually prevented by promoting soluble sugar accumulation, and this strategy increases tolerance to abiotic stress (Marcílio et al., 2019). In the current study, increment in sugar content exerts positive effects on human health from a nutrition perspective (Clara et al., 2018; Murali et al., 2020).

In the present study, the highest content of starch was detected in the post-blanching samples, and V11E0119, V11E0003, and V11E0012 showed significant high starch contents. Sun and Liu (2008) showed that blanching increases starch content in water dropwort. Similarly, Wu et al. (2006) mentioned that blanching enhances starch content in *Protea cynaroides*. Increases in the starch contents of blanched samples might be due to amyloplast formation and reduction in plant metabolism provoked by the dark conditions (Polesi et al., 2019). Another reason can be the downregulated expression of *greening after extended darkenss1* (*ged1*) and *prt6-1* genes, which facilitate starch storage and contribute to survival under stress conditions (Tamang and Fukao, 2015). Starch is of great value to human health and serves as a key source of carbohydrates in a balanced diet. Therefore, increased starch content in the blanched stem of water dropwort has a favorable effect on nutritional value.

Noctor et al. (1998) reported that amino acids content in poplar was greatly influenced by light conditions, and a comparatively low amino acid level was observed under dark conditions. In the current study, results show that total amino acids content in water dropwort was affected by blanching, consistent with previous findings (Noctor et al., 1998; Toldi et al., 2019). Furthermore, we found that the effect of blanching on V11E0003 and V11E0012 was minimal. Toldi et al. (2019) reported that the total content of amino acids decreased under low light conditions, and the ratio of serine family decreased by 50%. Moreover, low light can inhibit the expression of genes related to the metabolism of different amino acids, ultimately reducing the contents of amino acids (Toldi et al., 2019).

The shading treatments performed by Wang et al. (2020) considerably reduced total polyphenol and flavonoid contents, indicating that high light intensity plays an important role in the biosynthesis of phenols and flavonoids. In the present study, blanching markedly reduced polyphenol and flavonoid contents in all the cultivars of water dropwort, and the lowest contents were observed in the post-blanching samples. Moreover, blanching had minimal effects on V11E0100 and V11E0012 possibly because of light conditions that affected the polyphenols and flavonoids biosynthesis, and this variation also depends on the species and cultivars (Shao, 2010; Song et al., 2017). The low rate total phenols biosynthesis under lower light intensity is due to the reduced activity of the enzyme phenylalanine ammonia lyase (PAL) in the phenolic acid synthesis pathway (Kumari et al., 2009).

In plants, the contents of phenolic compounds are closely linked to their antioxidant activities (Sarker and Oba, 2018a, 2019d; Sarker et al., 2018a). Abiotic stress enhances the production of compounds with excellent redox properties by controlling the overproduction of reactive oxygen species (Sharma et al., 2012; Sarker and Oba, 2018a,d). In the current study, DPPH scavenging activity slightly increased in the stems of all the cultivars after the blanching period compared with that in the pre-blanching samples, and V11E0119 and V11E0012 showed higher DPPH scavenging activities. Similarly, the total antioxidant capacities of water dropwort cultivars during blanching increased significantly. The post-blanching samples of V11E0012 and V11E0100 showed the highest antioxidant capacities. Li and Pan (2018) showed that DPPH and total antioxidant activities in on Chinese water chestnut increase under etiolation. Similarly, Samuoliene et al. (2011) mentioned that DPPH activity increases under dark conditions because of the increased contents of phenols, vitamin C, and alpha-tocopherol.

In the present study, despite the decreases in the contents of polyphenols and flavonoids, antioxidant activity still increased because of the non-enzymatic antioxidant system composed of soluble sugars, vitamin C (ascorbic acid), vitamin B1 (thiamine), vitamin B2 (riboflavin), β-carotene (precursor of vitamin A), and α-tocopherol. These compounds have antioxidant properties and help reduce the effects of reactive oxygen species (Sharma et al., 2012; Deng et al., 2014; Padmanabhan et al., 2016; Subki et al., 2018; Soares et al., 2019). Currently, the blanched stem of water dropwort showed high antioxidant activities, which might have positive effects on human health.

Wu et al. (2007) mentioned that etiolated seedlings provide dietary fibers, vitamins, and phytonutrients. In the current study, we found high contents of vitamins A, B1, B2, and C during the blanching period, and higher contents were found present in the post-blanching samples. Moreover, the post-blanching samples of V11E0012 and V11E0100 showed the highest levels of these vitamins. Vitamin B1 increases under light, cold, salinity, and osmotic stress (Tunc-Ozdemir et al., 2009). A high level of vitamin B2 minimizes the adverse effects of stress, and the vitamin acts as an antioxidant (Deng et al., 2014). According to Hameed et al. (2005), the content of ascorbic acid considerably increases under etiolation in wheat seedlings, and the vitamin acts as an antioxidant to regulate defense under oxidative stress. Similarly, dark conditions increases the level of vitamin C and α-tocopherol (Samuoliene et al., 2011). Wu et al. (2007) reported that β-carotene content increases under etiolation and is a precursor of vitamins and antioxidants for humans. In the current study, high levels of these vitamins increase the nutritional value of blanched water dropwort and might help in the growth, development, and reproduction of the plant. The vitamins might also reduce the onset of diseases and improve health conditions (FAO, 2001; Insel and Roth, 2013). Therefore, we may assume that the high contents of these vitamins in blanched water dropwort contribute to the prevention or alleviation of diseases.

Different environmental factors influence the accumulation of minerals in plants. One of the main factors is light intensity, which affects the accumulation of minerals (Paiva et al., 2017; Sarker and Oba, 2018e). Colonna et al. (2016) observed that low light intensity induces the accumulation of K, Ca, and Mg in 10 leafy vegetables. Similarly, K, Mg, Ca, Zn Fe, and Mn levels are high in lettuce under low light intensity (Stagnari et al., 2015). Mineral content in ferns is inversely proportional to light intensity. Na, K, Mg, Ca, Mn, Fe, Cu, Zn, and total mineral contents considerably increase with decreasing light intensity (Wang et al., 2020). In the current study, we found that Na, Mg, Ca, Fe, and Mn contents increased during the blanching period, and the accumulation of these minerals was observed in the post-blanching samples. By contrast, K, Cu, and Zn level decreased during blanching. K and Na compete with each other because of their chemical similarities (Ahanger et al., 2017). Therefore, the accumulation of Na causes a decrease in the uptake rate of K in plants. Overall, minerals have highest degree of accumulation in V11E0012, followed by those in V11E0100 under blanching conditions. The variations in mineral uptake elucidates that blanching stimulates the minerals accumulation, which is consistent with the results of Lefsrud et al. (2006), Stagnari et al. (2015), and Wang et al. (2020). The reasons can be the stimulation of photosynthesis by high light intensity and decrease in mineral contents (Wang et al., 2020). Blanching has a positive effect on mineral accumulation, which is beneficial for consumer health because minerals have important roles in the growth and development of the human body. These minerals facilitate the formation of hemoglobin, bones, teeth, and proteins and promote nerve impulse and blood clotting. Furthermore, they are use in energy transfer and activate many enzymes (FAO, 2001; Cosmulescu et al., 2009; Insel and Roth, 2013). Similarly, few minerals, such as zinc, act as antioxidants, and iron and magnesium are cofactors for antioxidant activity and minimize the negative effect of free radicals on the human body (Kurtoglu et al., 2003; Prasad, 2014; Castellanos-Gutiérrez et al., 2018). Therefore, we speculate that these minerals in water dropwort can improve human health.

## Conclusion

We concluded that blanching considerably influences the nutrient level of water dropwort. Nutritional parameters, such as starch, sugars, vitamins, minerals, and antioxidant activities markedly increased in the blanched water dropwort stems. Furthermore, V11E0012 was found better than the other four cultivars in terms of nutritional profile. Therefore, blanched water dropwort can be used for achieving more nutrients and antioxidants, and the cultivar V11E0012 can be used for blanching cultivation.

## Data Availability Statement

The raw data supporting the conclusions of this article will be made available by the authors, without undue reservation.

## Author Contributions

HH and WK: conceptualization. JY and WK: funding acquisition. SK: data curation, investigation, and writing—original draft. SK, XH, QJ, and KZ: methodology. SK and GL: validation. SK, GL, JY, and HH: writing—review and editing. All authors have read and agreed to the published version of the manuscript and contributed to the manuscript.

## Conflict of Interest

The authors declare that the research was conducted in the absence of any commercial or financial relationships that could be construed as a potential conflict of interest.
