# Corrigendum: Phenotypic, Nutritional, and Antioxidant Characterization of Blanched *Oenanthe javanica* for Preferable Cultivar

**DOI:** 10.3389/fpls.2021.754226

**Published:** 2021-09-08

**Authors:** Sunjeet Kumar, Gaojie Li, Xinfang Huang, Qun Ji, Kai Zhou, Hongwei Hou, Weidong Ke, Jingjing Yang

**Affiliations:** ^1^The State Key Laboratory of Freshwater Ecology and Biotechnology, The Key Laboratory of Aquatic Biodiversity and Conservation of Chinese Academy of Sciences, Institute of Hydrobiology, Chinese Academy of Sciences, Wuhan, China; ^2^University of Chinese Academy of Sciences, Beijing, China; ^3^Institute of Vegetables, Wuhan Academy of Agricultural Sciences, Wuhan, China

**Keywords:** *Oenanthe javanica*, blanching, nutritional value, vitamins, minerals, Antioxidant capacity (AOC)

There is an error in the Funding statement. The correct number for the National Key R&D Program of China is 2020YFD0900305.

The authors apologize for this error and state that this does not change the scientific conclusions of the article in any way. The original article has been updated.

## Publisher's Note

All claims expressed in this article are solely those of the authors and do not necessarily represent those of their affiliated organizations, or those of the publisher, the editors and the reviewers. Any product that may be evaluated in this article, or claim that may be made by its manufacturer, is not guaranteed or endorsed by the publisher.

