# Supplementary material for: Phenotypic, Nutritional, and Antioxidant Characterization of Blanched Oenanthe javanica for Preferable Cultivar
Source: Front Plant Sci. 2021 Feb 19;12:639639. doi: 10.3389/fpls.2021.639639 (PMC7933200; doi:10.3389/fpls.2021.639639)
Supplement: Supplementary file 1 [file Image_1.pdf]

Supplementary Figure 1

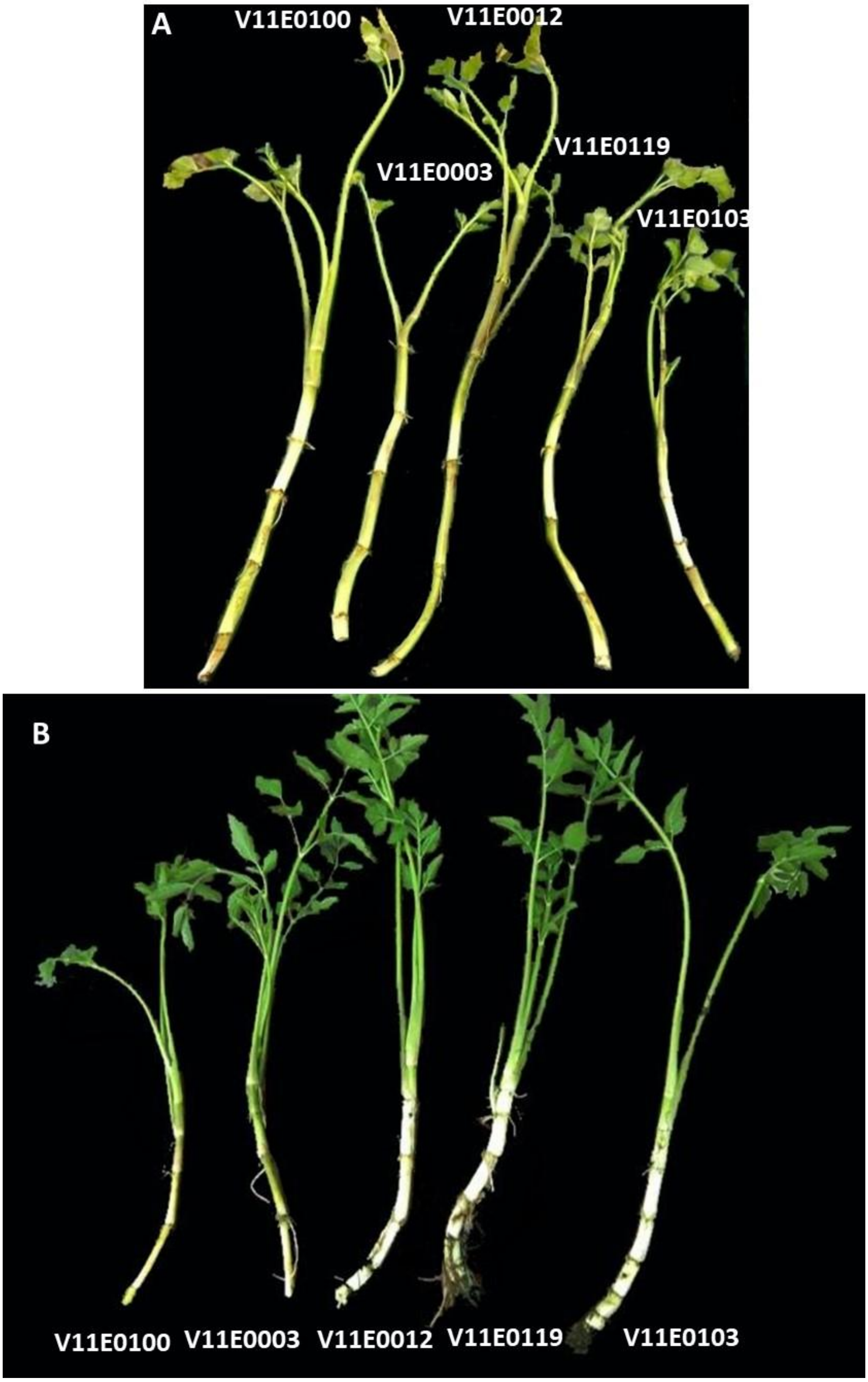

Figure S1 | Effect of blanching on the five cultivars of water dropwort. A) Effect of mid-blanching (after 20 days), and B) effect of post-blanching on the water dropwort (after 40 days).
